# Alcohol Misuse and Injury Outcomes in Young People Aged 10–24

**DOI:** 10.1016/j.jadohealth.2017.10.003

**Published:** 2018-04

**Authors:** Louise Lester, Ruth Baker, Carol Coupland, Elizabeth Orton

**Affiliations:** Division of Primary Care, School of Medicine, University of Nottingham, Nottingham, United Kingdom

**Keywords:** Alcohols, Hospitalization, Adolescent, Risk, Wounds and injuries

## Abstract

**Purpose:**

The burden of alcohol-attributable disease is a global problem. Young people often present to emergency health-care services with alcohol intoxication but little is known about how best to intervene at that point to improve future health outcomes. This study aimed to assess whether young people with an alcohol-specific hospital admission are at increased risk of injury following discharge.

**Methods:**

A cohort study was conducted using a general population of 10- to 24-year-olds identified using primary care medical records with linked hospital admission records between 1998 and 2013. Exposed individuals had an alcohol-specific admission. Unexposed individuals did not and were frequency matched by age (±5 years) and general practice (ratio 10:1). Incidence rates of injury-related hospital admission post discharge were calculated, and hazard ratios (HR) were estimated by Cox regression.

**Results:**

The cohort comprised 11,042 exposed and 110,656 unexposed individuals with 4,944 injury-related admissions during follow-up (2,092 in exposed). Injury rates were six times higher in those with a prior alcohol admission (73.92 per 1,000 person-years, 95% confidence interval (CI) 70.82–77.16 vs. 12.36, 11.91–12.81). The risk of an injury admission was highest in the month following an alcohol-specific admission (adjusted HR = 15.62, 95% CI 14.08–17.34), and remained higher compared to those with no previous alcohol-specific admission at 1 year (HR 5.28 (95% CI 4.97–5.60)) and throughout follow-up.

**Conclusions:**

Young people with an alcohol-specific admission are at increased risk of subsequent injury requiring hospitalization, especially immediately post discharge, indicating a need for prompt intervention as soon as alcohol misuse behaviors are identified.

**Implications and Contribution**

This study uses linked population-based datasets to describe the association between alcohol-specific hospital admission and subsequent risk of injury-related hospital admission in adolescents aged 10–24. With the greatest risk in the month after an alcohol admission, evidenced-based primary and secondary injury prevention and harm reduction programs should be implemented.

Alt-text: Unlabelled box

Globally, alcohol has been estimated to cause 3.3 million deaths per year, representing 5.9% of all deaths in 2012, and 5.1% of the global burden of disease [1]. Hazardous and harmful drinking is on the rise in young people [2], [3].

There are various adverse consequences of alcohol consumption and intoxication reported in young people. Acute impacts include depression, sleep disturbance, appetite change, reduced performance at school, crime, sexually transmitted infections, unwanted pregnancy, and mental health problems [4], [5], [6], [7], as well as significantly higher engagement in multiple risk behavior, including physical inactivity, self-harm, unprotected intercourse, and substance misuse [8]. In addition, a limited number of studies have shown that there is an increased risk of self-reported injury, repeated, and medically treated injury in young people who drink alcohol excessively [9], [10], [11], [12], [13], [14], and an association between heavy alcohol consumption in adolescence and increased injury risk in adulthood [15], [16], [17]. However, the detailed epidemiology of this relationship has not been described fully.

A small number of population-based cohort studies using hospital admission data from the United Kingdom have found that 10- to 19-year olds discharged from hospital after an adversity-related injury admission (related to violence, drugs/alcohol, or self-inflicted injury) have an increased risk of recurrent injury-related emergency admissions [18], subsequent re-admission, and death for up to a decade later, compared to those who had an accidental injury admission [19]. However, whether this type of association exists in cohorts of young people admitted into hospital because of excessive alcohol consumption is unclear.

Hospital admission resulting from excessive alcohol use provides a “teachable moment” for health promotion advice and the introduction of preventative interventions [18], [20]. However, current interventions are fairly generic, focusing on alcohol use behaviors rather than the prevention of specific outcomes related to alcohol. By describing alcohol-related injury risk in more detail, by for example, age, sex, and socioeconomic status, we can then tailor interventions more appropriately and potentially increase their efficacy.

The aim of this study, therefore, was to determine whether having an alcohol-specific hospital admission is associated with a higher rate of subsequent hospital admission for injury and to describe in detail how this varies by, age, sex, and socioeconomic deprivation, and over time in a population-based cohort of young people aged 10–24 in the United Kingdom.

## Methods

### Data sources

Two population-based health databases from the United Kingdom were utilized in this study; the Clinical Practice Research Datalink (CPRD) and linked Hospital Episode Statistics (HES). The CPRD [21] is one of the largest primary care research databases in the world, containing records from over 11.3 million patients [22]. Approximately 6.9% of the UK population is included in the database, and as over 98% of the UK resident population is registered with a primary care general practitioner [23], the data are broadly representative of the age, sex, and ethnicity profile of the whole population [22], [24]. The quality of CPRD data is subject to internal data quality checks, validation, audits, and up-to-standard requirements [24], [25], [26].

HES contains details of all hospital admissions and outpatient appointments at National Health Service hospitals and trusts in the United Kingdom, processing over 125 million records each year [27]. Each admission to hospital is coded using the International Statistical Classification of Diseases and Related Health Problems 10th Revision (ICD-10) with one primary diagnosis and up to 19 secondary diagnoses. Although HES contains admission and outpatient data, for this study, only hospital admission data were used to define both the exposure and the outcome. Linked HES-CPRD data are presently only available for English practices in CPRD who have consented to participate in the linkage scheme (398 of the 684 in the July 2014 CPRD release).

### Study population

**Figure 1:**
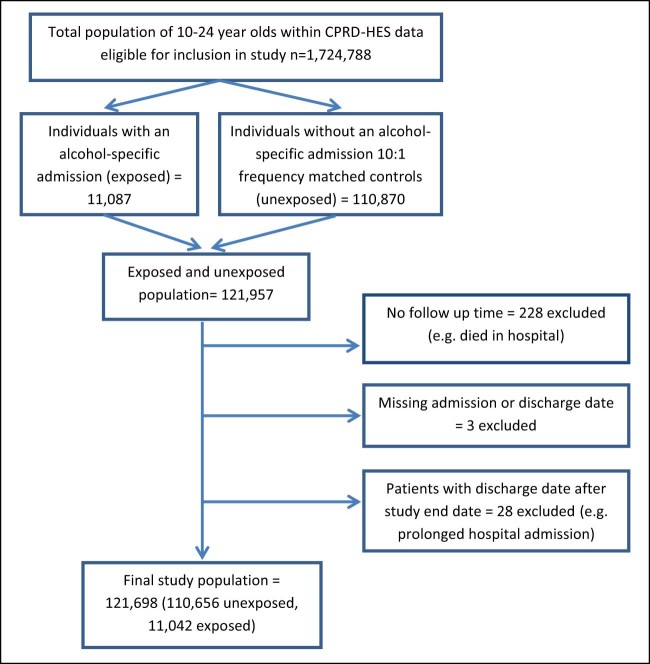
Study population flow chart.

The study population consisted of young people aged 10–24, registered at a CPRD practice in the United Kingdom between January 1, 1998 and December 31, 2013, who had linked HES data available and had data that met CPRD data quality standards. Young people initially entered the cohort at the latest of their 10th birthday or registration with a CPRD practice and were followed up until the earliest of either their 25th birthday, death, leaving their general practitioner's practice, or the practice's last data collection date. Young people who died on or after their alcohol admission date, had an invalid discharge date (e.g., before admission), or had no follow-up time were excluded from the analysis (Figure 1).

### Exposed group

Young people in the cohort who had an “alcohol-specific” hospital admission between the ages of 10 and 24 years were identified as the exposed group using an ICD-10 code list (Supplementary Table S1). An “alcohol-specific” admission is one in which the medical record included at least one ICD-10 code considered by Public Health England to be wholly attributed to alcohol (i.e., alcohol is 100% contributory as defined by an alcohol attributable fraction of 1.0) [28]. This code could appear in the primary or secondary diagnoses fields for the admission and individuals may have had other, concurrent, diagnosis codes at the time of admission. The first admission with an alcohol-specific diagnosis after cohort entry was used to define the date of admission, with exposed person-time starting at the date of “discharge” after that hospital admission.

### Selection of unexposed comparison group

A sample of young people in the cohort who had not had an alcohol-specific hospital admission between the ages of 10 and 24 was selected as an unexposed comparison group. Ten unexposed controls were frequency-matched to each exposed case. Frequency matching matches groups of subjects rather than individuals, ensuring both groups had the same age (in 5-year age bands) and registered GP practice distribution. Unexposed controls were assigned a “pseudo-event” date, which was a randomly generated date between cohort entry and exit dates.

### Outcome definition

The primary outcome was defined as the first record of a hospital admission with a primary or secondary diagnosis of injury within HES at least 1 day after the alcohol admission discharge/pseudo-event. ICD-10 injury codes included injury types S00–T98 and external causes (mechanisms, e.g., falls, transport, drowning/submersion) V01–Y98. If injury admissions were coded with more than one mechanism, a hierarchy was applied, adapting an existing framework [29] (Supplementary Table S2).

### Confounders

Age at alcohol admission/pseudo admission, sex, region of residence, calendar year, and socioeconomic deprivation were included as possible confounders. Age was defined at the date of alcohol-specific hospital discharge for those exposed or pseudo-event date for those unexposed. Geographical region was examined using the regional variable within CPRD (based on Strategic Health Authority administrative areas). Socioeconomic deprivation was measured using quintiles of the Index of Multiple Deprivation based on the individual's residential postcode.

### Analysis

Descriptive analyses were conducted to describe the demographics of the study population, reporting medians for age at exposure and follow-up time and proportions for all categorical variables. Chi-squared and Wilcoxon rank-sum tests were used to assess significant differences in characteristics between exposed and unexposed groups depending on the distribution of the data.

Crude incidence rates of the injury outcome (per 1,000 person-years), incidence rate ratios (IRRs), and 95% confidence intervals (CIs) were estimated overall and by sex, age at alcohol admission/pseudo admission date, and deprivation quintile. Hazard ratios (HRs) and 95% CIs for injury admission were estimated, comparing young people who had an alcohol-specific hospital admission and those who had not, using Cox regression analysis.

Potential confounders were tested by adding them to the model one at a time using a forward stepwise model, with likelihood ratio tests (LRT) conducted to assess if they should be included in the final multivariate model. Those variables found to be significant (*p* < .05) were included, with any previous nonsignificant confounders re-considered using LRTs to see if they remained nonsignificant. Interactions for age at alcohol admission/pseudo-admission date and sex were explored based on theoretical plausibility [18], [19] and were added as interaction terms into the models using LRTs to assess significance (using *p* < .01 to determine inclusion in the final model to account for the large sample size).

The proportional hazards assumptions were assessed by observing Kaplan-Meier and log-minus-log plots and using tests based on Schoenfeld residuals. Where these indicated the assumptions were not met, an interaction term between time and exposure was included in the model and HRs over time were calculated, with the log time interaction term providing the best fit to the data based on the Akaike information criterion.

### Subgroup analysis

A subgroup analysis was conducted restricting the definition of the exposure to only include those where the primary diagnoses for admission was alcohol (i.e., excluding those with a secondary diagnosis of alcohol).

### Ethical approval

Approval was obtained from the Independent Scientific Advisory Committee of the CPRD for protocol number 15_175. As CPRD data are anonymized, no additional National Health Service ethical approval was required.

## Results

### Cohort characteristics

There was a total of 121,698 young people aged 10–24 included in the cohort, who were registered at 388 GP practices with linked HES data from across the United Kingdom and contributed a total of 259,093 person-years of follow-up time. Of these, 11,042 had an alcohol-specific hospital admission and 110,656 were unexposed (Table 1).

**Table 1:** Characteristics of the cohort population, those with an alcohol-specific admission and frequency matched unexposed controls with no recorded alcohol-specific admission

Characteristic	Exposed: alcohol admission (n = 11,042)	Unexposed: no alcohol admission (n = 110,656)	*p* Value
**Follow-up after alcohol admission discharge or pseudo-admission date (y)**			<.0001^a^
Median (IQR)	2.17 (.82, 4.44)	1.20 (.38, 3.09)	
**Age at admission/pseudo admission (y)**			<.0001^a^
Median (IQR)	19.27 (16.28, 22.01)	19.29 (16.39, 22.86)	
**Sex n (%)**			<.0001^b^
Male	6,275 (56.83)	52,331 (47.29)	
Female	4,767 (43.17)	58,325 (52.71)	
**Region n (%)**			1.000^b^
North East	428 (3.88)	4,294 (3.88)	
North West	2,969 (26.89)	29,740 (26.88)	
Yorkshire and the Humber	492 (4.46)	4,921 (4.45)	
East Midlands	347 (3.14)	3,478 (3.14)	
West Midlands	1,277 (11.56)	12,789 (11.56)	
East of England	874 (7.92)	8,778 (7.93)	
South West	1,516 (13.73)	15,208 (13.74)	
South Central	1,136 (10.29)	11,378 (10.28)	
London	845 (7.65)	8,467 (7.65)	
South East Coast	1,158 (10.49)	11,603 (10.49)	
**Deprivation quintiles n (%)**			<.001^b^
1 (Least deprived)	1,418 (12.84)	18,103 (16.36)	
2	1,723 (15.60)	20,306 (18.35)	
3	1,986 (17.99)	20,934 (18.92)	
4	2,624 (23.76)	24,071 (21.75)	
5 (Most deprived)	3,274 (29.65)	26,990 (24.39)	
Missing	17 (.15)	252 (.23)	

IQR, interquartile range.

a Wilcoxon rank-sum test.

b Chi-squared test.

Those with an alcohol-specific admission were more likely to have longer follow-up time post alcohol admission (median 2.17 years vs. 1.20 years), be in the most deprived quintile (29.7% vs. 24.3%), and be male (56.8% vs. 47.3%) compared to those without an alcohol-specific hospital admission (all *p* < .0001).

### Injury outcomes

Of the 11,042 young people with an alcohol admission, 2,092 (18.9%) were admitted for an injury during study follow-up. This compared to 2,852 (2.6%) of the unexposed group (Table 2). Ninety-three percent of injury admissions had a mechanism code recorded (4616/4944). The most common three injury mechanisms in the exposed group were poisoning (n = 920, 44% of injury admissions), inanimate mechanical forces (n = 319, 15%), and animate mechanical forces (n = 237, 11%). In the unexposed group, the most common mechanisms were falls (n = 535, 19%), inanimate mechanical forces (n = 521, 18%), and poisoning (n = 511, 18%) (Supplementary Table S3).

**Table 2:** Crude injury rates for any injury outcome for those exposed (with a previous alcohol-specific hospital admission) and those unexposed (without)

Characteristic	Unexposed (no alcohol-specific admission)			Exposed (previous alcohol-specific admission)			Incidence rate ratio exposed-unexposed (95% CI)
	Person-years at risk (py)	No. of injury events (n)	Injury rate per 1,000 py (95% CI)	Person-years at risk (py)	No. of injury events (n)	Injury rate per 1,000 py (95% CI)	
**Total**	230,801.55	2,852	12.36 (11.91–12.82)	28,291.80	2,092	73.94 (70.84–77.18)	6.08 (5.75–6.43)
**Sex**							
Male	117,919.39	1,921	16.29 (15.58–17.04)	16,246.80	1,274	78.42 (74.23–82.84)	4.86 (4.53–5.22)
Female	112,882.16	931	8.25 (7.73–8.79)	12,045.01	818	67.91 (63.41–72.73)	8.50 (7.75–9.32)
**Age at alcohol admission**							
10–16	104,049.33	1,287	12.37 (11.71–13.06)	13,919.84	601	43.18 (39.86–46.77)	3.54 (3.21–3.90)
17–24	126,752.22	1,565	12.35 (11.75–12.97)	14,371.96	1,491	103.74 (98.61–109.15)	8.46 (7.89–9.08)
**Deprivation quintile**							
1 (Least deprived)	38,559.12	352	9.13 (8.22–10.13)	3,914.25	213	54.42 (47.58–62.24)	6.09 (5.15–7.19)
2	43,723.49	477	10.91 (9.97–11.94)	4,647.78	266	57.23 (50.75–64.54)	5.27 (4.55–6.11)
3	43,650.80	514	11.78 (10.80–12.84)	5,072.72	347	68.41 (61.57–76.00)	5.90 (5.16–6.75)
4	50,250.16	647	12.88 (11.92–13.91)	6,761.39	501	74.10 (67.89–80.88)	5.85 (5.22–6.57)
5 (Most deprived)	54,219.73	858	15.82 (14.80–16.92)	7,865.30	760	96.63 (90.00–103.75)	6.20 (5.63–6.84)
Missing	398.24	4	10.04 (3.77–26.76)	30.37	5	164.65 (68.53–395.57)	21.02 (4.62–95.65)

CI, confidence interval.

Table 2 shows crude incidence rates for the injury admission outcome for all individuals and by sex, age at alcohol admission/pseudo admission, and deprivation quintile. The injury incidence rate was six times higher in the exposed group at 73.94 per 1,000 person-years compared to 12.36 per 1,000 person-years in unexposed young people. This increased risk between those exposed and unexposed was highest in females (IRR 8.50, 95% CI 7.75–9.32), the older age group (IRR 8.46, 95% CI 7.89–9.08 for ages 17–24), and those in the most deprived quintile (6.20, 95% CI 5.63–6.84).

### Hazard ratios

**Figure 2:**
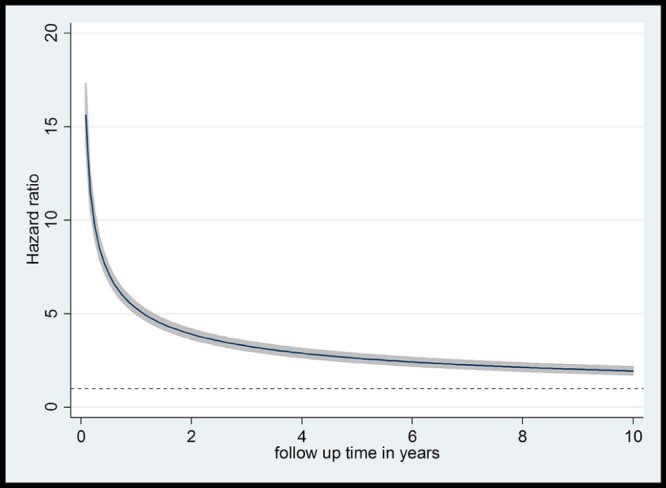
Hazard ratios with 95% CI for any injury admission over time following an alcohol-specific admission.

The unadjusted HR for first subsequent injury admission was 6.22 (95% CI 5.88–6.59) for those in the exposed group compared to the unexposed group overall (Figure 2). There was no significant interaction between age at alcohol admission/pseudo admission and sex (*p* = .75). The proportional hazards assumption was not met in the Cox model for the alcohol admission exposure with a significant LRT for the interaction term between follow-up time and exposure (*p* < .001), indicating that the risk of injury changes over time following exposure. Table 3 shows adjusted hazard ratios for the model including an interaction term between exposure and log follow-up time and adjusting for age at alcohol admission/pseudo admission, deprivation, region, and sex. The interaction term (.65, 95%CI .62–.67 per unit increase in log (time)) indicates this risk depreciates significantly over time (Figure 2). For example, the risk of admission for injury was highest in the first month after exposure, with the exposed young people having a 15.6 times higher risk of injury admission at 1 month (HR = 15.62, 95% CI 14.08–17.34). At 6 months this had reduced to 7.14 times (95% CI 6.71–7.60), at 1 year it was 5.3 times higher (HR 5.28, 95% CI 4.97–5.60), and at 5 years 2.61 times higher (95% CI 2.39–2.86).

**Table 3:** Adjusted hazard ratios for a subsequent injury admission to hospital

Characteristic	Hazard ratio (95% CI)
**Alcohol admission (yes/no) evaluated at 1 year's follow-up**	5.28 (4.97–5.60)
**Interaction term for alcohol admission and log (follow-up time)**	.65 (.62–.67)
**Sex**	
Male	1.00
Female	.64 (.60–.67)
**Age at alcohol admission (per year)**	1.02 (1.01–1.03)
**Deprivation quintile**	
1 (Least deprived)	1.00
2	1.13 (1.01–1.26)
3	1.24 (1.12–1.38)
4	1.35 (1.22–1.49)
5 (Most deprived)	1.63 (1.47–1.80)
Missing	1.43 (.74–2.77)
**Region**	
North East	1.00
North West	.98 (.85–1.11)
Yorkshire and the Humber	.91 (.76–1.08)
East Midlands	.89 (.73–1.09)
West Midlands	.86 (.74–1.00)
East of England	.83 (.70–.98)
South West	.94 (.82–1.10)
South Central	.86 (.73–1.01)
London	.72 (.60–0.85)
South East Coast	.87 (.74–1.02)

### Subgroup analysis

The exposed group fell into two categories: those with an alcohol-specific primary diagnosis (n = 3,739) (Supplementary Table S5) and those with an alcohol-specific secondary diagnosis (n = 7,303) (Supplementary Table S6). Of those with a secondary diagnosis that was alcohol-specific, the most common primary diagnoses were related to injury and poisoning. There were a total of 41,061 young people aged 10–24 included in the subgroup analysis restricted to those with a primary diagnosis of alcohol; 3,739 exposed (34% of all those with an alcohol admission) and 37,322 matched controls. Similar to the main analysis, exposed individuals had a significantly higher risk of a subsequent injury admission compared to those who had not had an alcohol-specific admission. The hazard ratio at 1 year was lower than in the main analysis (HR 3.96, 95% CI 3.58–4.37 compared to HR 5.28, 95% CI 4.97–5.60), but is still a significant increase in injury admission risk of four times compared to those with no alcohol-specific admission.

The most common injury mechanisms were similar to those in the main analysis; for the exposed group, poisonings were the most common (n = 221, 36%), and for those unexposed, falls were the most frequent mechanism recorded in the admission (n = 237, 21%) (Supplementary Table S4).

## Discussion

### Summary of main findings

This large cohort study has identified a significant association between an alcohol-specific hospital admission between the ages of 10and 24 and subsequent risk of injury-related hospital admission. We found that individuals with a previous alcohol admission were 6 times more likely to have a subsequent injury admission, with an absolute increase in injury rate of 61.6/1,000 person-years. The relative increases in injury rate were greatest for females, those in the older age group (17–24 years), and in the most deprived quintile. In addition, the risk of injury admission was 15 times higher in the first month after an alcohol admission, remaining five times higher compared to those with no previous alcohol-specific admission at 1 year.

An important finding was that 82% of young people in the exposed group, who had an alcohol-specific diagnosis as a secondary diagnosis, had a primary diagnosis of either poisoning or injury. This suggests there may be a number of different etiological groups of young people who experience alcohol-specific hospital admissions. For example, those who misuse alcohol on its own as a one-off, those who have an alcohol misuse issue and attend repeatedly, those with concurrent alcohol and injury/poisoning admissions, and those with concurrent alcohol and mental health-related admissions. Further research is needed to investigate this hypothesis.

### Strengths and limitations

This is one of the largest studies worldwide to describe the association between alcohol misuse and subsequent injury risk in young people, using a large population-based cohort with results therefore generalizable. A key strength of our study was the use of non–self-reported exposure and outcome measures, limiting associated recall and response biases. Previous studies reporting an association have largely been cross-sectional, such that temporal relationships cannot be confirmed and were often restricted to specific ages. Both these and the limited cohort studies available to date have used mostly self-reported exposure and outcome measures, often single survey questions, asked on a single day [10], [11], [12], [13], [14], [17], [30].

There are, however, several potential limitations of our study and areas for further investigation. The exposure classification may have been affected by under-recording (e.g., not recognizing/recording alcohol involvement) or incorrect coding of alcohol misuse using ICD-10 codes [31]. There may also have been some young people misclassified as unexposed who had an alcohol-specific admission prior to entering the cohort (e.g., moved from a non-CPRD practice). However, this misclassification is likely to dilute results and lead to an underestimation of the effect size in this study. Also related to the exposure was our finding that those admitted with a secondary diagnosis of alcohol harm often had a concurrent record of poisoning. This may have been a bias introduced due to clinical policy. In the United Kingdom, since 2004, the National Institute of Health and Care Excellence recommends young people presenting with self-harm be admitted overnight [32]. This may have increased the proportion of young people in our exposed group at risk of subsequent self–harm-related injury. This is important since young people who self-harm are susceptible to repeat harm events [33]. The large number of poisoning injuries observed in the exposed group during the initial alcohol-specific and subsequent admission may represent young people repeatedly self-harming [34]. However, without examining intent, it is difficult to ascertain if subsequent poisonings are related to repeated self-harm or substance misuse (including alcohol), or both. This warrants further investigation.

If there was a concurrent injury at the time of the exposure admission, it is possible that some of the subsequent injury hospital admissions were for follow-up care rather than being new incident events. However, it is likely most of these would be treated within primary care or outpatients and would not be considered as a new hospital admission. Furthermore, previous research suggests that a poisoning admission occurring greater than 1 week from hospital discharge is likely to be a new poisoning injury event [35]; therefore, the majority of admissions are unlikely to be related to follow-up care.

Young people who attend the emergency department with alcohol-specific diagnoses and injuries are not necessarily admitted into in-patient wards. Since emergency department data were not linked to the CPRD at the time of this study, we were unable to include these. Likewise, injuries only presented to primary care were not included in our study. This was because the focus of the study was to assess secondary health-care burden and also because injury mechanism is not well recorded in primary care data [35]. Therefore, utilizing only hospital admissions is an important limitation of our study, with those included in this study potentially representing a very specific group of young people, likely to be at the severe end of alcohol misuse and injury requiring admission, or where there are concerns about intentional injury.

Finally, follow-up time was short, although greater in those exposed with a previous alcohol-specific admission compared to those unexposed in this study. Follow-up in our cohort may be impacted by the move in residency of adolescents of this age (e.g., moving away to university) and therefore GP practice.

### Comparison to the literature

There are few population-based cohorts that examine the association between alcohol misuse and subsequent injury outcomes in this age group, so direct comparisons are limited. In existing cohort studies [15], [16], the risk of subsequent injury is also associated with a previous alcohol admission (odds ratios 1.4–1.74). However, as we report HRs, and these previous studies report odds ratios, our results are not directly comparable.

The most comparable findings are from population-based cohort studies by Herbert et al. [18], [19] utilizing the United Kingdom HES data. They report that HRs for death and emergency readmission were higher at 10 years following a previous adversity-related injury admission (violent, drug/alcohol-related, or self-inflicted injury) in 10- to 19-year-olds. This suggests a consistent pattern to our results for injury admission following an alcohol-specific admission; although our study includes young people up to age 24 and focuses on injury-related readmissions following an alcohol-specific admission as opposed to the broader definitions used by Herbert et al. Therefore, it may be the alcohol component of the exposure that has the greatest risk, or the injury outcome that is most likely.

### Implications for research

This study suggests the relationship between a young person's first alcohol-specific hospital admission and injury outcomes is complex. We have highlighted potential subgroups of adolescents who are admitted to hospital with alcohol-specific causes, some with potentially ongoing mental health problems. This needs further investigation to understand the relationship better.

The further use of linked data (emergency attendance data and primary care records) could provide a wider definition of both the alcohol exposure and subsequent injury outcomes, capturing young people at the less severe end of the spectrum and also including information on different groups of young people (e.g., those with comorbidities) [13]. This would be useful in informing future policy, especially if subsequent injury risk is increased following a lower alcohol misuse threshold.

Further studies could examine in greater detail the mechanism, intent, and type of injuries most likely to occur following a previous alcohol-specific admission and the risk of these different injuries. This would support a greater understanding of the population at risk and enable targeting of programs.

### Implications for practice

The findings of our study have implications for those involved in the commissioning and provision of injury prevention and harm reduction programs for young people and specialist alcohol services. As the greatest risk of injury is in the month following the alcohol-specific admission, early interventions during the admission and/or discharge process, taking account of intent, are indicated for these young people, which capitalize on the teachable moment opportunity. Our study suggests programs may need tailoring to the group of young people involved. For example, given the high proportion of poisoning injury admissions in young people with a previous alcohol-specific hospital admission, there may be implications for specialist primary and secondary interventions involving child and adolescent mental health services for certain groups. The feasibility, effectiveness, and cost-effectiveness of injury prevention programs targeted at different groups of young people with an alcohol-specific hospital admission should be evidenced before such programs can be commissioned on a wider scale. However, this study provides a foundation for potential injury prevention policy and practice changes for young people who misuse alcohol.
