# Supplementary material for: Alcohol Misuse and Injury Outcomes in Young People Aged 10–24
Source: J Adolesc Health. 2018 Apr;62(4):450–6. doi: 10.1016/j.jadohealth.2017.10.003 (PMC5861304; doi:10.1016/j.jadohealth.2017.10.003)
Supplement: Appendix S1 — Tables S1–S6. [file mmc1.docx]

**Supplementary table 1 - Alcohol-specific ICD-10 codes used to define exposure status**

| **ICD-10 code** | **ICD-10 description** |
| --- | --- |
| E244 | Alcohol-induced pseudo-Cushing's syndrome |
| F10 | Mental and behavioural disorders due to use of alcohol |
| F100 | Acute intoxication |
| F101 | Harmful use |
| F102 | Dependence syndrome |
| F103 | Withdrawal state |
| F104 | Withdrawal state with delirium |
| F105 | Psychotic disorder |
| F106 | Amnesic syndrome |
| F107 | Residual and late-onset psychotic disorder |
| F108 | Other mental and behavioural disorders |
| F109 | Unspecified mental and behavioural disorder |
| G312 | Degeneration of nervous system due to alcohol |
| G621 | Alcoholic polyneuropathy |
| G721 | Alcoholic myopathy |
| I426 | Alcoholic cardiomyopathy |
| K292 | Alcoholic gastritis |
| K70 | Alcoholic liver disease |
| K700 | Alcoholic fatty liver |
| K701 | Alcoholic hepatitis |
| K702 | Alcoholic fibrosis and sclerosis of liver |
| K703 | Alcoholic cirrhosis of liver |
| K704 | Alcoholic hepatic failure |
| K709 | Alcoholic liver disease, unspecified |
| K860 | Alcohol-induced chronic pancreatitis |
| T510 | Toxic effect: Ethanol |
| T511 | Toxic effect: Methanol |
| T519 | Toxic effect: Alcohol, unspecified |
| X45 | Accidental poisoning by and exposure to alcohol |
| X450 | Accidental poisoning by and exposure to alcohol |
| X451 | Accidental poisoning by and exposure to alcohol |
| X452 | Accidental poisoning by and exposure to alcohol |
| X453 | Accidental poisoning by and exposure to alcohol |
| X454 | Accidental poisoning by and exposure to alcohol |
| X455 | Accidental poisoning by and exposure to alcohol |
| X456 | Accidental poisoning by and exposure to alcohol |
| X457 | Accidental poisoning by and exposure to alcohol |
| X458 | Accidental poisoning by and exposure to alcohol |
| X459 | Accidental poisoning by and exposure to alcohol |

**Supplementary table 2 – Injury ICD-10 codes used to define the outcome by mechanism hierarchy**

| **Mechanism** | **ICD-10 codes** | **Hierarchy level^a^** |
| --- | --- | --- |
| Transport | V01-V99, X82-X829, Y03-Y039, Y32-Y329, | 1 |
| Falls | W00-W199, X80-X809, Y01-Y019, Y30-Y309 | 2 |
| Animate mechanical forces | W50-W649, Y04-Y059 | 3 |
| Inanimate mechanical forces | W20-W499, X72-X759, X78-X799, X93-X969, X99-Y009, Y22-Y259, Y28-Y299, Y350-Y351, Y353-Y354, Y360-y362 | 4 |
| Smoke, fire | X00-X099, X76-X769, X97-X979, Y26-&269, Y363 | 5 |
| Heat and hot substances | X10-X199, X77-X779, X98-X989, Y27-Y279 | 6 |
| Threats to breathing | W75-W849, X70-X709, X91-X919, Y20-Y209 | 7 |
| Drowning/submersion | W65-W749, X71-X719, X92-X929, Y21-Y219 | 8 |
| Poisoning | X40-X499, X60-X699, X85- X909, Y10-Y199, Y352 | 9 |
| Electric current, radiation | W85-W999 | 10 |
| Travel, overexertion | X50-X579 | 11 |
| Venomous animals/plants | X20-X299 | 12 |
| Forces nature | X30-X399 | 13 |
| Other, specified | X58-X589, X81-X819, X83-X839, Y02-Y029, Y06-Y089, Y31-Y319, Y33-Y339, Y35, Y355-y36, Y364-y369 | 14 |
| Other, unspecified | X59-X599, X84-X849, Y09-Y099, Y34-Y349, | 15 |
| Supplementary factors | Y90-798 | 16 |

**^a^** *Hierarchy level used to determine the underlying injury mechanism used for reporting mechanism proportions (i.e where an injury hospital admission has two or more mechanism codes the one highest in the hierarchy is used).*

Supplementary table 3 - Injury mechanism of subsequent injury admissions, by exposed and unexposed groups for main analysis

| **Injury mechanism n (%)** | **Total population: all injury admission patients (n= 4, 944)** | **Exposed: previous alcohol admission (n=2,092)** | **Unexposed: no previous alcohol admission (n=2,852)** |
| --- | --- | --- | --- |
| Transport  Falls  Animate mechanical forces  Inanimate mechanical forces  Smoke, fire  Heat &hot substances  Threats to breathing  Drowning/submersion  Poisoning  Electric current, radiation  Travel, overexertion  Venomous animals/plants  Forces of nature  Other, specified  Other, unspecified  Supplementary factors  Not recorded | 513 (10.38)  741 (14.99)  617 (12.48)  840 (16.99)  23 (0.47)  15 (0.30)  21 (0.42)  3 (0.06)  1,431 (28.94)  6 (0.12)  53 (1.07)  2 (0.04)  4 (0.08)  48 (0.97)  281 (5.68)  17 (0.34)  329 (6.65) | 106 (5.07)  206 (9.85)  237 (11.33)  319 (15.25)  7 (0.33)  7 (0.33)  8 (0.38)  1 (0.05)  920 (43.98)  2 (0.10)  11 (0.53)  0 (0.00)  2 (0.10)  18 (0.86)  93 (4.45)  11 (0.53)  144 (6.88) | 407 (14.27)  535 (18.76)  380 (13.32)  521 (18.27)  16 (0.56)  8 (0.28)  13 (0.46)  2 (0.07)  511 (17.92)  4 (0.14)  42 (1.47)  2 (0.07)  2 (0.07)  30 (1.05)  188 (6.59) 6 (0.21)  185 (6.49) |

Supplementary table 4 - Injury mechanisms for the subgroup cohort with a subsequent injury admission, by exposed and unexposed groups

| **Injury mechanism n (%)** | **Total population: all injury admission patients (n=1,759 )** | **Exposed: previous alcohol admission (n=612)** | **Unexposed: no previous alcohol admission (n=1,147)** |
| --- | --- | --- | --- |
| Transport  Falls  Animate mechanical forces  Inanimate mechanical forces  Smoke, fire  Heat &hot substances  Threats to breathing  Drowning/submersion  Poisoning  Electric current, radiation  Travel, overexertion  Venomous animals/plants  Forces of nature  Other, specified  Other, unspecified  Supplementary factors  Not recorded | 221 (12.56)  314 (17.85)  224 (12.73)  290 (16.49)  10 (0.57)  3 (0.17)  5 (0.28)  1 (0.06)  409 (23.25)  2 (0.11)  26 (1.48)  1 (0.06)  3 (0.17)  22 (1.25)  113 (6.42)  9 (0.51)  106 (6.03) | 47 (7.68)  77 (12.58)  74 (12.09)  104 (16.99)  3 (0.49)  2 (0.33)  3 (0.49)  0 (0.00)  221 (36.11)  2 (0.33)  4 (0.65)  0 (0.00)  2 (0.33)  7 (1.14)  34 (5.56)  5 (0.82)  27 (4.41) | 174 (15.17)  237 (20.66)  150 (13.08)  186 (16.22)  7 (0.61)  1 (0.09)  2 (0.17)  1 (0.09)  188 (16.39)  0 (0.00)  22 (1.92)  1 (0.09)  1 (0.09)  15 (1.31)  79 (6.89)  4 (0.35)  79 (6.89) |

**Supplementary table 5 - Frequency of alcohol-specific ICD-10 codes for admissions where the primary diagnosis was an alcohol-specific cause (n=3,739 admissions)**

| **ICD-10 code** | **ICD-10 description** | **Frequency (%)** |
| --- | --- | --- |
| F10.0 | Mental and behavioural disorders due to use of alcohol, acute intoxication | 2,937 (78.6) |
| T51.0 | Toxic effect: Ethanol | 245 (6.6) |
| F10.1 | Mental and behavioural disorders due to use of alcohol, harmful use | 146 (3.9) |
| T51.9 | Toxic effect: Alcohol, unspecified | 119 (3.2) |
| F10.2 | Mental and behavioural disorders due to use of alcohol, dependence syndrome | 84 (2.3) |
| K29.2 | Alcoholic gastritis | 69 (1.9) |
| F10.3 | Mental and behavioural disorders due to use of alcohol, withdrawal state | 51 (1.4) |
| F10.9 | Mental and behavioural disorders due to use of alcohol, Unspecified mental and behavioural disorder | 26 (0.7) |
| K86.0 | Alcohol-induced chronic pancreatitis | 18 (0.5) |
| F10.5 | Mental and behavioural disorders due to use of alcohol, Psychotic disorder | 17 (0.5) |
| K70.1 | Alcoholic hepatitis | 10 (0.3) |
| K70.9 | Alcoholic liver disease, unspecified | 6 (0.2) |
| F10.4 | Mental and behavioural disorders due to use of alcohol, Withdrawal state with delirium | <5 (0.1) |
| T51.1 | Toxic effect: Methanol | <5 (0.1) |
| F10.6 | Mental and behavioural disorders due to use of alcohol, Amnesic syndrome | <5 (<0.1) |
| F10.8 | Mental and behavioural disorders due to use of alcohol, Other mental and behavioural disorders | <5 (<0.1) |
| K70.2 | Alcoholic fibrosis and sclerosis of liver | <5 (<0.1) |
| K70.3 | Alcoholic cirrhosis of liver | <5 (<0.1) |

**Supplementary table 6 - Primary diagnosis for hospital admission where an alcohol-specific cause was a secondary diagnosis (n=7,303 admissions)**

| **ICD-10 code** | **ICD-10 description** | **Frequency (%)** |
| --- | --- | --- |
| T36-T65 | Poisoning / toxic effect of substances | 3,316 (45.4) |
| S00-T36, T66-T98, V01-X59 | Injury | 2,682 (36.7) |
| R00-R99 | Symptoms, signs and abnormal clinical/lab findings, (e.g. chest pain, palpitations, syncope, abnormal LFTs) | 472 (6.5) |
| K00-K93 | Diseases of the digestive system | 240 (3.3) |
| F00-F99 | Mental and behavioural disorders | 202 (2.8) |
| E00-E90 | Endocrine, nutritional and metabolic diseases | 68 (0.9) |
| G00-G99 | Diseases of the nervous system | 58 (0.8) |
| O00-O99 | Pregnancy, childbirth and the puerperium | 45 (0.6) |
| I00-I99 | Diseases of the circulatory system | 41 (0.6) |
| Z00-Z99 | Factors influencing health status and contact with health services | 40 (0.6) |
| M00-M99 | Diseases of the MSK system and connective tissue | 37 (0.5) |
| J00-J99 | Diseases of the respiratory system | 32 (0.4) |
| N00-N99 | Diseases of the genitourinary system | 25 (0.3) |
| A00-B99 | Infectious and parasitic diseases | 15 (0.2) |
| L00-L99 | Diseases of the skin and subcutaneous tissue | 9 (0.1) |
| - | Other | 21 |
